# **Modeling thermoelectric performance of p-type Cu**_**3**_ **SbSe**_**4**_ **-based chalcogenide materials using decision trees and structural risk error minimization intelligent computational methods**

**DOI:** 10.1371/journal.pone.0339521

**Published:** 2026-01-20

**Authors:** Fawaz Saad Alharbi

**Affiliations:** Department of Mechanical Engineering, College of Engineering, University of Hafr Al Batin, Hafr Al Batin, Saudi Arabia; Universidad Especializada de las Americas, PANAMA

## Abstract

Cu_3_SbSe_4_-based materials are ternary chalcogenides thermoelectric compounds with unique sphalerite super-lattice structures and adjustable characteristics which stand them out as promising material for attaining efficient thermal and electrical energy conversion. The crystal structure of Cu_3_SbSe_4_-based materials consists of Cu-Se three dimensional frameworks with inserted CuSe_4_ tetrahedra layer. This energy band structure and crystal arrangement in Cu_3_SbSe_4_-based materials lead to large seebeck coefficient, low thermal conductivity and large carrier mobility with restricted number of available carriers which hinders the potential of these materials as thermoelectric compound due to low value of thermoelectric performance. Experimental methods of thermoelectric performance (using figure of merit as a measure of energy conversion efficiency) enhancement are laborious, costly and consume appreciable resources which necessitate the need of computational methods for figure of merit prediction. In this contribution, figure of merit of Cu_3_SbSe_4_-based materials has been modeled through random forest regression (decision trees) and genetic algorithm incorporated support vector regression (structural risk error minimization-based) model using temperature, dopants ionic radii and their respective concentrations as predictors. Genetically optimized support vector regression (GESVR) model outperforms random forest regression (RFR)-based model with improvement of 188.04%, 30.18% and 42.36% using correlation coefficient, mean absolute error and root mean square error, respectively for testing samples of Cu_3_SbSe_4_-based compounds. Influence of inclusions on energy conversion efficiency of Cu_3_Sb_1-x_Sn_x_Se_4_ and Cu_3_Sb_1-x_Fe_x_Se_2.8_S_1.2_ compounds was investigated using GESVR- based model. The simplicity of descriptors coupled with the demonstrated precision would facilitate the exploration of Cu_3_SbSe_4_-based materials for green applications and ultimately address the current global energy crisis.

## 1. Introduction

Thermoelectric materials are potential materials that can address the global energy crisis through conversion of wasted heat energy directly into electricity without the associated mechanical vibrations, light and noise [1–3]. Aside from addressing environmental and energy crisis, thermoelectric materials remain promising source of clean electricity [4,5]. The potential and strength of thermoelectric materials for energy conversion is evaluated using parameter (dimensionless) known as figure of merit [6–8]. Figure of merit is computed through electrical conductivity, Seebeck coefficient, total thermal conductivity and absolute kelvin temperature. Unfortunately, optimizing thermal and electrical conductivities simultaneously seems tedious due to the existence of inverse dependence between the two conductivities. Many research work in this area aims at developing materials with huge energy conversion efficiency [9–12]. In an effort to attain high conversion efficiency in thermoelectric materials, nanostructured materials have characteristic quantum confinement effect and thermal conductivity induced due to boundary scattering, which respectively translate to high Seebeck coefficient and low thermal conductivity with ultimate improvement in thermoelectric figure of merit [13].Thermoelectric performance enhancement through nano structuring and band gap structure optimization has been reported [14]. Filtering of electrons with characteristic low energy at the interface further strengthens thermal conversion efficiency in nanostructured materials. However, physical methods of bulk quantity synthesis such as melt spinning and ball-milling are time consuming, energy intensive and costly. Furthermore, majority of thermoelectric materials with better energy conversion efficiency are expensive, toxic and/ or contain rare elements. Recently, significant attention has been drawn into thermoelectric materials with characteristic features of low toxicity, low cost and earth abundance [15,16]. Copper nano-particles have been reported to have diverse applications in different fields [17]. Similarly, chalcogenide based materials play useful roles in several optical applications [18]. However, Cu-based materials satisfy these environmental friendliness, abundance and cheap conditions and have attracted attentions. Copper based chalcogenides compounds hold promising potentials in energy conversion technology and have characteristic potentials of structural stability and environmental friendliness [19,20]. Cu_3_SbSe_4_ thermoelectric material addresses major challenges associated with classical thermoelectric materials like Bi_2_Te_3_ (that contains tellurium which is very scarce and costly), PbTe (that contains Pb which is environmentally hazardous) and other tellurium based materials [21]. Thermoelectric figure of merit of Cu_3_SbSe_4_ class of copper-based chalcogenide materials is modeled in this work using temperature and molecular properties of the material as the descriptors.

Remarkable thermoelectric features demonstrated by Cu_3_SbSe_4_ (copper based chalcogenides) material strengthen their potentials in addressing energy crisis through conversion of heat to electricity and vice versa using the principle of Seebeck and Peltier effect [22,23]. Cu_3_SbSe_4_-based compounds are p-type ternary semiconductors with zinc blend-like tetragonal structure and ZnSe origin as revealed by XRD and XRF spectroscopy [24,25]. Crystal structural description reveals two Cu atoms (not equivalent) while four Se atoms serve as the nearest neighbors to each Sb and Cu atoms in the crystal structure. Hence, there exist slightly flattened tetrahedral (SbSe_4_) and distorted tetrahedral (CuSe_4_) crystallographic architecture [24]. Therefore, the slightly flattened tetrahedral part constitutes three-dimensional Cu/Se frameworks while the hybridization of Cu3d-Se4p (resulted from distorted tetrahedral part) provides Cu/Se framework with high mobility. This promotes low phonon conductivity due to the increase in phonon-phonon interactions [9]. The intrinsic features of the space group associated with these compounds couple with narrow band gap and large effective mass translate to low thermal conductivity and large Seebeck coefficient which can ultimately strengthen the energy conversion efficiency [26]. However, limited concentration of carriers restricts the value of figure of merit to lower values. This serves as the bottleneck for their large-scale production for many industrial and technological applications. Research effort concentrates on carrier concentration enhancement through dopants incorporation at Cu-site, Sb-site and/or Se- site. Inclusion of second phase with higher concentration of carriers remains additional strategy of figure of merit enhancement. Defect introduction with different sizes potentially strengthen phonon scattering with energy conversion efficiency improvement [27]. However, experimental methods of thermoelectric figure of merit enhancement through doping, second phase and defect inclusion are laborious, costly and consume appreciable resources which necessitate the need of computational methods for addressing the challenges. This work presents intelligent models for figure of merit determination through hybrid genetic algorithm-based support vector regression and random forest regression (decision tree based) algorithms using temperature and molecular properties as the descriptors.

Support Vector Regression (SVR) is a structural risk based computational technique utilized for predicting continuous target variables [28,29]. SVR is rooted in the concepts of Support Vector Machines (SVMs), which were initially developed for classification tasks. In the case of regression, SVR seeks to find a function which approximates the connection within input features and target variable while maintaining a robust margin of error [30–32]. The core idea behind SVR is to transfer data samples into new space using kernels, thereby allowing the algorithm to capture complex relationships in the data that traditional linear regression models might miss. Once in the feature space, SVR searches for a hyper-plane that minimizes the deviation between the estimated and measured values, subject to a margin of tolerance [33]. This approach enables SVR to effectively handle both linear and non-linear regression problems. A key advantage of SVR over conventional regression models is its ability to model non-linear relationships without the need for explicitly specifying the nature of non-linearity. Through the use of kernels, such as the Gaussian and polynomial kernels, SVR can operate in a high-dimensional space without having to compute the transformation explicitly, making it computationally efficient. SVR based model has been reported to preserve its accuracy and precision when developed using relatively few data samples [34], which necessitate its choice in modeling thermoelectric performance of Cu_3_SbSe_4_-based compounds due to limited available experimental data samples for model development and validation. SVR model is particularly effective in situations where the relationship between input features and the target is complex such as thermoelectric performance modeling of copper-based chalcogenides. In predicting the thermoelectric performance of Cu_3_SbSe_4_-based materials, hyper-parameters associated with SVR algorithm which include the epsilon, penalty factor and mapping parameter, were optimized through genetic algorithm. Genetic Algorithm (GA) is an optimization technique inspired by Charles Darwin’s theory of natural evolution, which proposes that the best solutions to a problem evolve over successive generations [35,36]. GA simulates the process of natural selection, where problem optimization evolves through selection, crossover, and mutation to generate improved solutions [37]. The prediction capacity of genetically optimized support vector regression (GESVR) model was compared with random forest regression (RFR) based model using various assessment parameters. RFR is a sophisticated ensemble learning technique that constructs a “forest” of decision trees, aggregating their predictions to enhance robustness and predictive accuracy [38]. Each decision tree is built using bootstrap sampling, which involves selecting random subsets of variables at every split. Assessment parameters for performance evaluation include correlation coefficient (CC), mean absolute error (MAE) and root mean square error (RMSE).

Performance comparison between GESVR and RFR model shows superiority of GESVR over RFR model using MAE, CC and RMSE computed through training and testing samples of Cu_3_SbSe_4_ materials. Performance enhancement of 3.38% and 188.04% was attained by GESVR over RFR model for training and testing Cu_3_SbSe_4_-based compounds, respectively using CC performance metric. Using MAE assessment parameter, GESVR demonstrates improvement over RFR model for the respective set of Cu_3_SbSe_4_-based samples with superiority of 83.43% and 30.18%. Similarly, enhancements of 85.45% and 42.36% were respectively obtained using RMSE metric. Figure of merit enhancement potential of tin (Sn) and iron (Fe) particles inclusion in Cu_3_Sb_1-x_Sn_x_Se_4_ and _Cu3Sb1-x_Fe_x_Se_2.8_S_1.2_ compounds were modeled and investigated using GESVR model.

The rest of the manuscript is arranged and organized as follows: section two presents the mathematical review and foundation of support vector regression algorithm as well as genetic algorithm. Section two further details the operational principle of random forest regression algorithm. Computational formulation and details of the hybrid intelligent algorithm (GESVR) and RFR are presented and discussed in section three. Description of Cu_3_SbSe_4_ materials with the governing empirical relation is presented in section three. The fourth section discusses the predicted figure of merit for Cu_3_SbSe_4_-based compounds and their comparisons using different performance metrics. The dependence of figure of merit on dopants concentration was simulated using GESVR model. Section five contains the summary of model outcomes.

## 2. Mathematical foundation of the intelligent algorithms

This section presents the formulation of structural risk error minimization-based model (GESVR), genetic algorithm and random forest regression.

### 2.1. Support vector regression intelligent algorithm

For modeling thermoelectric figure of merit of Cu_3_SbSe_4_-based compounds, Support Vector Regression (SVR) applies the function described in Equation (1) to effectively model and forecast the underlying patterns and dependencies within thermoelectric figure of merit, temperature, ionic radii of the incorporated dopants and their associated concentrations [39,40].


μ(b)=⟨w, b⟩+ϕ    (1)

Here, μ denotes the estimated figure of merit generated by the regression model, b are the input features which include temperature, ionic radii of the incorporated dopants and their associated concentrations. The weight vector (w) and bias (ϕ) coefficients, which are key components in the model, are determined through risk function minimization as by outlined in Equation (2).


$$\begin{array}{c} \left(\mu \right)=\frac{p}{m}\sum_{k=\text{1}}^{n}I\left(\mu (b)-\;\;\mu_{k}^{*}\right)+\;\frac{∥\beta {∥}^{\text{2}}}{\text{2}} \end{array}$$ (2)

From Equation (2), $∥\beta {∥}^{\text{2}}$ represents the Euclidean norm, while ***I*** define the error threshold (loss function) as shown in Equation (3) and $\mu^{*}$ represents the measured energy conversion efficiency. The regularization factor, denoted as ***p***, imposes a penalty for deviations beyond the epsilon threshold [41].


$$I\left(\mu^{*}\;,\mu (b)\;\right)=\;\;\left\{ \begin{array}{c} \text{0}\;\;\;\;\;\;\;\;\;\;\;\;\;\;\;\;\;if\;|\mu^{*}-\mu (b)|\leq \varepsilon \\ \left|\mu^{*}-\mu (b)\right|-\varepsilon \;,otherwise \end{array}\right.\;\;\;$$ (3)

The inclusion of slack variables becomes crucial, especially when there exists the possibility of exceeding the defined error threshold. Slack variables help control the distance between the observed figure of merit and the defined boundaries. The optimization process, addressing this dual problem, is executed through the method described in Equation (4) and is subject to the constraints set forth in Equation (5).


$$\begin{array}{c} Minimize:\;\frac{\text{1}}{\text{2}}\sum_{k}^{n}\sum_{i}^{n}\left(\varepsilon_{k}-\varepsilon_{k}^{*}\right)\left(\varepsilon_{i}-\varepsilon_{i}^{*}\right)\lambda \left(b_{k},\;b_{i}\right)+\sum_{k=\text{1}}^{n}\mu_{k}^{*}\left(\varepsilon_{k}-\varepsilon_{k}^{*}\right)-\sigma \sum_{k=\text{1}}^{n}\left(\varepsilon_{k}+\varepsilon_{k}^{*}\right)\;\; \end{array}$$ (4)

$$\begin{array}{c} \sum_{k=\text{1}}^{n}\left(\varepsilon_{k}+\varepsilon_{k}^{*}\right)=\text{0},\varepsilon_{k},\varepsilon_{k}^{*}\;\epsilon [\text{0},p]\;\;\; \end{array}$$ (5)

Where, $\varepsilon_{k}$ and $\varepsilon_{k}^{*}$ stand for the Lagrange multipliers used for optimization. The data points associated with non-zero coefficients in the solution are referred to as support vectors. Equation (6) represents the estimated figure of merit generated by the algorithm, leveraging the optimization approach previously discussed.


$$\begin{array}{c} \mu (b)=\sum_{k=\text{1}}^{n}\left(\varepsilon_{k}-\varepsilon_{k}^{*}\right)\left\langle b_{k},\;b\right\rangle +\phi \;\;\; \end{array}$$ (6)

The kernel function plays a pivotal role in converting the original non-linear function into a linear one within a space with higher dimensionality. This transformation is captured by the mapping function outlined in Equation (7), while the final form of the non-linear regression model in the SVR framework is depicted by Equation (8).


$$\gamma \left(b_{k},b_{i}\right)=\;\varphi \left(b_{k}\right)\varphi \left(b_{i}\right)\;\;\;$$ (7)

$$\begin{array}{c} \mu (b)=\sum_{k=\text{1}}^{n}\left(\varepsilon_{k}-\varepsilon_{k}^{*}\right)\gamma \left(b_{k},b_{i}\right)+\phi \;\;\; \end{array}$$ (8)

### 2.2. Genetic population-based optimization algorithm

**Fig 1:**
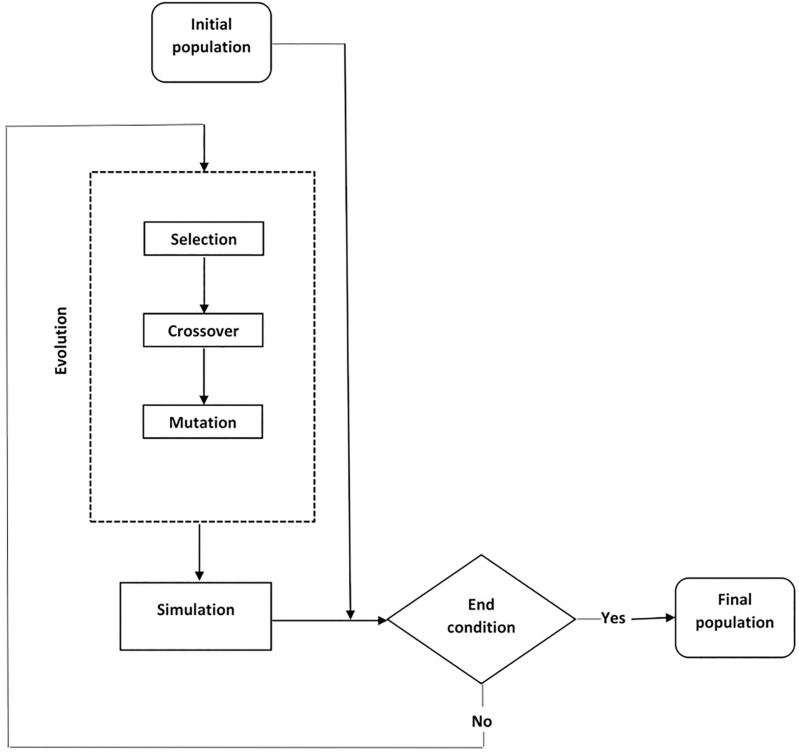
Computational flow chart of genetic algorithm for SVR parameters optimization.

Genetic algorithm implementation procedures and processes begin with the initialization of a population of chromosomes while the chromosomes are the probable solution of the optimization problem [42,43]. These chromosomes are evaluated using a fitness function that quantifies how well they solve the problem. Solutions with higher fitness scores have higher chance of been involved in reproduction stage, leading to the creation of subsequent generation. Through the processes of selection, crossover, and mutation, the population evolves iteratively, with the aim of finding the optimal solution [44]. The selection process involves choosing individuals based on their fitness scores, with better solutions having a higher probability of being selected. Crossover, or recombination, utilizes solutions of the two parents for offspring generation that may inherit the best features of both parents. Mutation introduces small random changes to an individual’s genes, ensuring genetic diversity and preventing premature convergence to suboptimal solutions [45]. The evolution process continues until a predefined termination condition is met, such as reaching a maximum number of generations or achieving an acceptable fitness level. Genetic algorithm effectively addresses complex optimization problems where traditional methods may struggle, such as in high-dimensional or non-linear optimization spaces. **Fig 1** illustrates the typical flow of the genetic algorithm.

### 2.3. Random forest decision tree algorithm

An ensemble learning method called Random Forest Regression (RFR) builds a “forest” of decision trees and aggregates their predictions to improve robustness and accuracy of the generated model [46,47]. Decision trees are created using bootstrap sampling, which selects a random selection of variables at each split and leaves some samples for validation. Diversity existing within decision trees is controlled with the incorporated randomness and thereby mitigates the tendency of the model to enter into over/under-fitting problem. The final estimates of RFR prediction come from aggregation and average of the predictions across all the decision trees [48]. Among the uniqueness associated with forest regression algorithm is the unique capacity in assessing and evaluating the significance as well as the possible contribution of each of the feature to the model precision and accuracy. Hence, inter-feature correlation is significantly minimized while using RFR algorithm. Equation (9) presents the estimated figure of merit {$\hat{ZT}(b\}$ for Cu_3_SbSe_4_-based compounds where $\hat{{ZT}_{i}}(b)$ is the figure of merit for each decision tree within the forest and J represents the total number of trees.


$$\begin{array}{c} \hat{ZT}(b)=\;\frac{\text{1}}{J}\sum_{i=\text{1}}^{J}\hat{{ZT}_{i}}(b) \end{array}$$ (9)

## 3. Computational methodology

Description of Cu_3_SbSe_4_-based data samples utilized for modeling and simulation is discussed and detailed in this section. Computational hybridization of genetic algorithm with support vector regression algorithm is further discussed. The section also contains the computational details of random forest algorithm for thermoelectric figure of merit prediction in Cu_3_SbSe_4_ system of materials.

### 3.1. Description and acquisition of Cu_3_SbSe_4_ data samples for modeling and simulation

The set of data samples employed for modeling figure of merit of Cu_3_SbSe_4_ system of materials consists of temperature, ionic radii of four different possible incorporated dopants and their respective concentrations. Measured thermoelectric figure of merit utilized for simulation was extracted from twenty-six different Cu_3_SbSe_4_ compounds reported in the literature [8–10,19,20,22–24,26,27,49–62]. Empirical relation presented in Equation (10) summarizes Cu_3_SbSe_4_ system of materials that can be modeled using the proposed GESVR and RFR algorithms in this work.


$$Cu_{\text{3}-x}\beta_{x}Sb_{\text{1}-y-\delta }\gamma_{y}\varphi_{\delta }Se_{\text{4}-\rho }\theta_{\rho }$$ (10)

Where β = inclusion at Cu-site, x = inclusion concentration at Cu-site,  γ = first inclusion at Sb-site, y = inclusion concentration at Sb-site, φ = second dopant at Sb-site,  δ = concentration of second inclusion at Sb-site, θ = inclusion at Se-site and ρ = inclusion concentration at Se-site. For example, if thermoelectric figure of merit of Cu_3_Sb_0.93_Mn_0.06_Sn_0.01_Se_4_ is to be predicted at a particular temperature using the proposed models in this work, the descriptors to the model would be defined as β = 0, x = 0,  γ = ionic radius of Mn, y = 0.06, φ = ionic radius of Sn,  δ = 0.01, θ = 0 ρ = 0. Statistical analysis of the employed data samples are shown in Table 1. FM and T respectively represent the thermoelectric figure of merit and the temperature. The statistical parameters computed include the mean, maximum, standard deviation, minimum and correlation coefficient for all input predictors and the corresponding figure of merit.

**Table 1:** Statistical report of data samples employed for figure of merit prediction in Cu_3_SbSe_4_ system of material.

Parameter	Mean	Maximum	Standard deviation	Correlation coefficient	Minimum
FM	0.7282	1.1800	0.2638	1.0000	0.0330
T (K)	629.1923	700.0000	61.1032	0.7015	380.0000
β	8.3135	108.0000	29.3469	0.3344	0.0000
x	4.1712	108.0000	21.1771	0.3285	0.0000
γ	81.0000	133.0000	34.1154	0.1119	0.0000
y	0.0324	0.0800	0.0232	0.1259	0.0000
φ	6.4615	85.0000	22.8285	0.0772	0.0000
δ	0.0027	0.0600	0.0119	0.0843	0.0000
θ	8.1923	111.0000	25.1221	0.1856	0.0000
ρ	0.0658	1.2000	0.2512	0.2080	0.0000

The computed mean values for all the predictors and experimental figure of merit provide the background information regarding the overall average of the data samples content. Standard deviations provide information regarding the consistency of the data samples as extracted from different experimental methods. Minimum and maximum values measure the range of each of the input features while the correlation dictates the extent of linear relation existing between the input features and the thermoelectric figure of merit. All input features show positive correlation with the figure of merit while the coefficients of correlation are relatively low except the temperature. This observation directly indicates the weakness of linear models in establishing the relationship. Hence, non-linear algorithm such as the one proposed in this work have potential in addressing the existing non-linear relationship existing between temperature, molecular descriptors and thermoelectric figure of merit for Cu_3_SbSe_4_ system of materials.

### 3.2. Hybridization details of genetic based support vector regression

**Fig 2:**
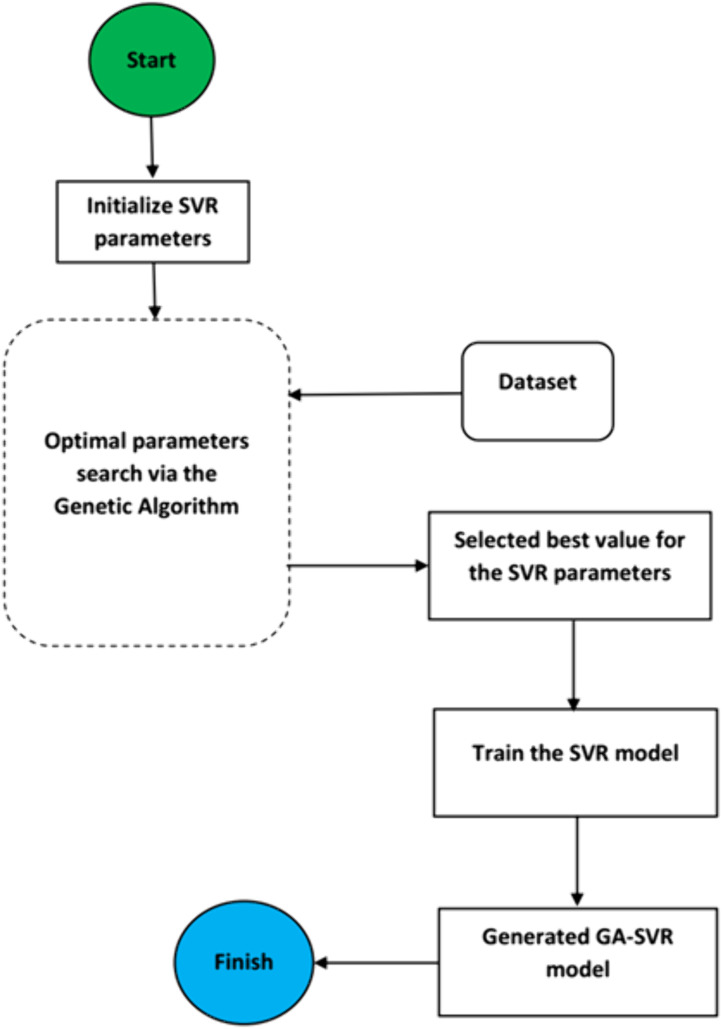
Computational flow chart of fitness computation using SVR algorithm.

In this study, genetic algorithm (GA) was combined with Support Vector Regression (SVR) algorithm to optimize key parameters of the SVR model, thus improving its predictive performance. This hybrid model leverages the strengths of both GA and SVR: GA provides an effective global search mechanism for tuning the hyper-parameters, while SVR efficiently handles the non-linear regression tasks. The methodology proceeds as follows: (a) *Data Preparation*: the dataset extracted from Cu_3_SbSe_4_ system of materials was first randomized to minimize bias and then split into training and testing sets with 80:20 ratio. This ensures that the model can be evaluated on unseen data samples to assess its generalization ability. (b) *Population Initialization*: The search space for key SVR parameters (C, epsilon, and kernel parameters) was defined. Chromosomes were initialized, with each chromosome representing a potential combination of hyper-parameters. The bounds for the parameters were set to [2000, 10] for penalty factor, [0.008, 0.02] for epsilon, and [0.008, 0.02] for the kernel parameter. (c) *Fitness Evaluation:* the fitness of each chromosome was evaluated by calculating the root mean square error (RMSE) on the testing Cu_3_SbSe_4_ system of materials. The SVR model was trained using the parameters specified by each chromosome, and the RMSE between the predicted and actual values was computed. The chromosomes with the lowest RMSE values were deemed to have better fitness. (d) *Selection:* chromosomes with higher fitness scores were selected for reproduction. A selection probability of 0.8 was used to choose parents for the next generation. (e) *Crossover:* the crossover operation combines two parent chromosomes to produce offspring by swapping segments of their genetic material. This process is controlled by a crossover probability of 0.9, ensuring that the offspring inherit the best traits from both parents. (f) *Mutation:* mutation was applied to introduce small random changes in the chromosomes, helping to maintain diversity in the population. This operation was performed with a low mutation probability of 0.005. (g) *Termination:* the algorithm was terminated when one of the following conditions was satisfied: the RMSE reached zero, the maximum number of generations was reached, or the RMSE value has remained unchanged for 50 consecutive generations. **Fig 2** presents the computational details for fitness computation.

### 3.3. Computational implementation of random forest regression algorithm with decision trees ensembles

Random forest regression algorithm was implemented for thermoelectric figure of merit prediction in Cu_3_SbSe_4_ system of materials using series of steps itemized in this section. Before the commencement of model development, samples extracted from Cu_3_SbSe_4_ system of materials were randomized and separated into training and testing set using 4:1 ratio. Cu_3_SbSe_4_ system of materials was subsequently subjected to the following procedures: (a) *Bootstrap sampling*: random selection of instances from training samples of Cu_3_SbSe_4_ system of materials with replacement constitutes bootstrap sampling. The decision trees within the defined forest were trained using these samples. (b) *Decision tree construction:* Iterative division of available thermoelectric data samples extracted from Cu_3_SbSe_4_ system of materials was utilized for decision trees development. Randomness was introduced by the random forest algorithm through selection of subset features at each node. This characteristic distinguishes random forest from the conventional decision tree which considers all features without reservation. The introduced randomness builds diversity in the system and minimizes possible correlation among the trees and thereby strengthens the prediction accuracy of the algorithm. (c) *Development of trained model:* each tree was trained using specific bootstrap sample. Therefore, the final model collects diverse and complementary decision trees. (d) *Thermoelectric figure of merit prediction for Cu*_*3*_*SbSe*_*4*_ *system of materials:* Every tree within the forest predicts thermoelectric figure of merit of Cu_3_SbSe_4_ system of materials while the final prediction combines the estimation from all the trees and compute the average values. (e) *Assessment of model performance*: the prediction precision of the developed model was assessed through testing samples of Cu_3_SbSe_4_ system of materials which were not included for model training. The assessment was carried out using MAE, CC and RMSE for both training and testing samples of Cu_3_SbSe_4_ system of materials. Cross validation approach employed allows definition of solution space for model parameters. The solution space for maximum features was defined as (2–4), space for minimum samples of leaf was defined as (1–5), space for minimum samples of split was set as (1–6) and number of estimator was defined as (20,35,50,70). The optimum values for the maximum depth, maximum features, minimum samples of leaf, minimum samples of split and number of estimators were obtained as 30, 2, 1, 5 and 35, respectively.

## 4. Results and discussion

The prediction outcomes of GESVR and RFR models are presented in this section together with their comparison. Discussion regarding hyper-parameter optimization using genetic algorithm was also taken into consideration. Effect of dopants inclusion on thermoelectric figure of merits of classes of Cu_3_SbSe_4_ system of materials was investigated and discussed in this section.

### 4.1. Convergence of GESVR parameters using genetic algorithm

**Fig 3:**
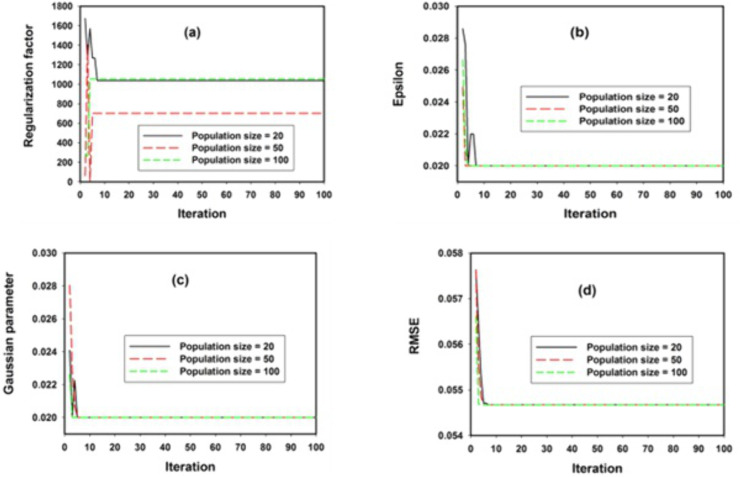
Convergence optimization of GESVR parameters (a) regularization factor (b) Epsilon threshold (c) Gaussian kernel factor (d) RMSE fitness parameter.

Convergence outcomes of optimization of support vector regression algorithm using genetic algorithm are presented in **Fig 3** for thermoelectric figure of merit prediction in Cu_3_SbSe_4_ system of materials.

Regularization factor which controls the penalty assigned to data-points outside error boundaries is shown in **Fig 3a** for different sizes of population ranges from twenty to one hundred chromosomes within the solution space. Twenty population size shows convergence towards higher values of regularization factors while increase in population size to fifty brings the regularization factor towards a lower value. Further increase in population size to a hundred further elevates the values of regularization factor. The optimum value of regularization factor was achieved at twenty numbers of chromosomes exploiting and exploring the solution space since the size leads to minimum error associated with thermoelectric figure of merit for Cu_3_SbSe_4_ system of materials. The epsilon convergence which controls the generalization training error bound is shown in **Fig 3b** at population sizes of twenty, fifty and hundred within the solution space. The observed convergence in this case is robust and relatively independent on the population size since similar convergence and behavior was obtained for different sizes of the chromosomes. **Fig 3c** presents the convergence for Gaussian mapping function parameter. Other mapping functions such as sigmoid and polynomial were investigated but only Gaussian has been reported due to its superior performance over other investigated mapping functions. Mapping function performs the role of data samples transformation from solution space to a new space with high degree of dimensionality where conventional regression was performed. Similarly, Gaussian parameter convergence shows robust convergence and independent on the chromosome size. Error convergence and chromosome fitness as measured using root mean square error (RMSE) is presented in **Fig 3d**. RMSE between the measured and predicted thermometric figure of merit does not change with the number of chromosomes exploring and exploiting the solution space. Table 2 contains the optimum values of the model parameters as obtained using genetic evolutionary population-based algorithm.

**Table 2:** Optimum parameters for thermoelectric figure of merit prediction in Cu_3_SbSe_4_ system of materials.

Model parameters	Optimum
Epsilon	0.02
Regularization factor	1037.901
Kernel function	Gaussian
Gaussian parameter	0.02
Population size	20

### 4.2. Evaluation and assessment of model performance using correlation and error metrics

**Fig 4:**
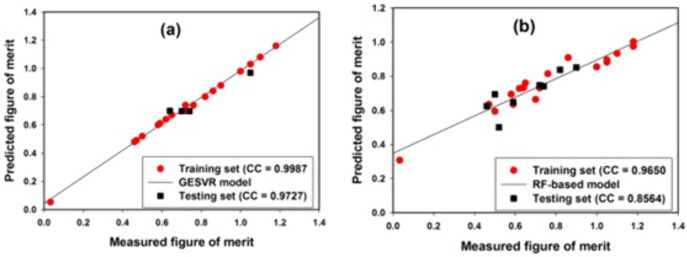
Cross-plots using correlation metric for (a) GESVR (b) RFR models using training and testing samples of Cu_3_SbSe_4_ system of materials.

The performance of the developed RFR and GESVR model was evaluated, assessed and computed using parameters such as correlation coefficient (CC), mean absolute error (MAE) and root mean square error (RMSE). **Fig 4** shows the correlation cross-plot between measured and estimated thermoelectric figure of merit for Cu_3_SbSe_4_ system of materials using GESVR and RFR models. For GESVR model, coefficient of 99.87% was obtained using the training samples of Cu_3_SbSe_4_ system of materials while the testing samples have associated correlation coefficient of 97.27% as shown in **Fig 4a**. However, correlation coefficients of 96.5% and 85.64% were respectively obtained for training and testing samples of Cu_3_SbSe_4_ system of materials presented in **Fig 4b** using RFR model. High value of correlation coefficient directly translates to significant alignment of data samples as can be generally observed in **Fig 4**. GESVR model that employs structural risk principle of error minimization demonstrates better performance as well as data sample alignment as compared with RFR model which employs ensemble of decision trees.

**Fig 5:**
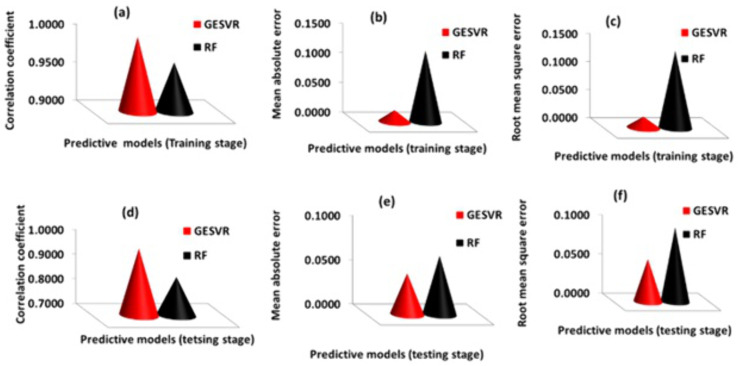
Performance comparison between GESVR and RFR models using (a) CC training (b) MAE training (c) RMSE training (d) CC testing (e) MAE testing and (f) RMSE testing of Cu_3_SbSe_4_ system of materials.

Performance comparison using varieties of metrics is shown in **Fig 5**. The metrics computed consist of MAE, CC and RMSE for training and testing compounds of Cu_3_SbSe_4_ system of materials.

**Fig 5a** presents the comparison using CC for training samples of Cu_3_SbSe_4_ system of materials. GESVR model shows CC of 99.87% while CC of 96.5% was computed for RFR model. Comparison using MAE presented in **Fig 5b** for training samples of Cu_3_SbSe_4_ system of materials indicates 0.02 and 0.12 for GESVR and RFR model, respectively for thermoelectric figure of merit prediction while **Fig 5c** shows the comparison for training samples of Cu_3_SbSe_4_ system of materials using RMSE metric with 0.02 and 0.14, respectively. **Fig 5d**, **5e** and **5f** respectively compares the two models (GESVR and RFR) for testing samples of Cu_3_SbSe_4_ system of materials using CC, MAE and RMSE metrics. Using GESVR and RFR model, 97.27% and 85.64% were respectively obtained using CC metric, 0.05 and 0.07 were respectively obtained using MAE metric and, 0.05 and 0.09 were respectively obtained using RMSE metric. Using percentage comparison approach, GESVR model outperforms RFR model for thermoelectric figure of merit prediction with respective improvement of 83.43%, 3.38% and 85.45% using MAE, CC and RMSE metrics, for training compounds of Cu_3_SbSe_4_ system of materials. For testing samples of Cu_3_SbSe_4_ system of materials, corresponding percentage improvement of 188.04%, 30.18% and 42.36%, were respectively obtained. Table 3 contains parameters with performance insights and the percentage improvement of GESVR model over RFR model for different samples of Cu_3_SbSe_4_ system of materials.

**Table 3:** Performance evaluation parameters for GESVR and RFR model with their percentage superiority using different samples of Cu_3_SbSe_4_ system of materials.

	Training Cu_3_SbSe_4_-based materials			Testing Cu_3_SbSe_4_-based materials		
	CC	MAE	RMSE	CC	MAE	RMSE
GESVR	0.9987	0.02	0.02	0.9727	0.05	0.05
RF	0.9650	0.12	0.14	0.8564	0.07	0.09
% improvement of GESVR over RF	3.38	83.43	85.45	188.04	30.18	42.36

### 4.3. Estimates of GESVR and RFR models and their associated absolute errors

The predictions of GESVR and RFR models are presented in Table 4 for all the investigated Cu_3_SbSe_4_ system of materials at different temperatures. The absolute error between the outcomes of the predictive models and the measured values are also presented. The predictions of GESVR agree with the reported thermoelectric figure of merits while the values predicted using RFR model show slight deviations. All thermoelectric figure of merit predicted by GESVR model has associated error of 0.02 except few compounds such as Cu_3_Sb_0.98_Sn_0.02_Se_4_ [60] with 0.08 deviation, Cu_3_Sb_0.93_Mn_0.06_Sn_0.01_Se_4_ [55] with 0.04 deviation, Cu_3_Sb_0.96_Sn_0.04_Se_4_ [19] with 0.06 deviation, Cu_3_Sb_0.96_Pb_0.04_Se_4_ [10] with maximum deviation of 0.18 and Cu_2.95_Sb_0.96_Ge_0.04_Se_4_ [24] without any deviation from the measured value. Similarly, RFR model estimates thermoelectric figure of merit of Cu_3_SbSe_4_ system of materials with deviation ranges between 0.27 for Cu_3_Sb_0.99_Pb_0.01_Se_4_ [56] and 0.0 for Cu_3_Sb_0.93_Mn_0.06_Sn_0.01_Se_4_ [55] compound.

**Table 4:** Estimated thermoelectric figure of merit for Cu_3_SbSe_4_ system of materials.

Compound	Measured	Temperature (K)	GESVR	Absolute error	RF	Absolute error
Cu_3_Sb_0.96_Pb_0.04_Se_4_	0.52 [8]	623	0.70	0.18	0.50	0.02
Cu_2.85_Ag_0.15_Sb_0.985_Bi_0.015_Se_4_	1.18 [41]	673	1.16	0.02	1.00	0.18
Cu_3_Sb_0.98_5Sn_0.015_Se_4_	1.00 [16]	623	0.98	0.02	0.85	0.15
Cu_3_Sb_0.91_Sn_0.03_Hf_0.06_Se_4_	0.82 [50]	623	0.80	0.02	0.84	0.02
Cu_3_Sb_0.96_Ti_0.04_Se_4_	0.59 [17]	623	0.61	0.02	0.65	0.06
Cu_3_Sb_0.92_Sc_0.08_Se_4_	0.47 [51]	623	0.49	0.02	0.63	0.16
Cu_3_Sb_0.92_Y_0.08_Se_4_	0.46 [51]	623	0.48	0.02	0.62	0.16
Cu_3_Sb_0.96_Sn_0.04_Se_4_	0.64 [14]	673	0.70	0.06	0.73	0.09
Cu_3_Sb_0.95_Fe_0.05_Se_2.8_S_1.2_	0.86 [52]	673	0.84	0.02	0.91	0.05
Cu_3_Sb_0.997_In_0.003_Se_4_	0.50 [53]	648	0.52	0.02	0.59	0.09
Cu_3_Sb_0.96_Co_0.04_Se_4_	0.62 [54]	673	0.64	0.02	0.73	0.11
Cu_3_Sb_0.96_Sn_0.04_Se_4_	0.72 [54]	673	0.74	0.02	0.73	0.01
Cu_3_Sb_0.93_Mn_0.06_Sn_0.01_Se_4_	0.74 [42]	673	0.70	0.04	0.74	0.00
Cu_3_Sb_0.99_Pb_0.01_Se_4_	0.03 [43]	380	0.05	0.02	0.31	0.27
Cu_3_Sb_0.98_Sn_0.02_Se_4_	1.05 [44]	623	1.03	0.02	0.88	0.17
Cu_2.95_Sb_0.96_Ge_0.04_Se_4_	0.70 [18]	640	0.70	0.00	0.66	0.04
Cu_2.98_SbSe_4_	0.59 [45]	650	0.61	0.02	0.64	0.05
Cu_3_Sb_0.98_Bi_0.02_Se_3.99_Te_0.01_	0.76 [6]	650	0.74	0.02	0.81	0.05
Cu_2.99_Ni_0.01_SbSe_4_	0.65 [46]	650	0.67	0.02	0.76	0.11
Cu_3_Sb_0.98_Sn_0.02_Se_4_	1.05 [47]	690	0.97	0.08	0.90	0.15
Cu_2.85_Ag_0.15_SbSe_4_	0.90 [20]	623	0.88	0.02	0.85	0.05
Cu_2.8_Ag_0.2_Sb_0.95_Sn_0.05_Se_4_	1.18 [19]	623	1.16	0.02	0.98	0.20
Cu_3_Sb_0.97_Al_0.03_Se_4_	0.58 [7]	600	0.60	0.02	0.69	0.11
Cu_3_Sb_0.94_Sn_0.06_Se_3.5_S_0.5_	1.10 [48]	700	1.08	0.02	0.93	0.17
Cu_3_Sb_0.95_Sn_0.05_S_4_	0.72 [49]	623	0.70	0.02	0.75	0.03
Cu_2.8_SbSe_4_	0.50 [15]	523	0.52	0.02	0.69	0.19
**Mean absolute deviation**				**0.03**		**0.10**

Mean absolute deviation for GESVR and RFR models are 0.03 and 0.1, respectively as shown in Table 4. This shows that the thermoelectric figure of merit predicted using GESVR model are closer to the measured values as compared with the outcomes of RFR model. Superior performance demonstrated by hybrid GESVR model can be attributed to the structural risk principle of error minimization utilized by the algorithm coupled with its strong mathematical formulation as compared with RFR model that operates using ensemble of decision trees.

### 4.4. Influence of dopants inclusion in crystal structure of Cu_3_SbSe_4_ system of materials using GESVR model

For further assessment of the predictive strength and generalization capacity of GESVR model, the model was employed for determining thermoelectric figure of merit of two copper-based chalcogenide compounds and presented in **Fig 5**. At this stage of modeling and simulation, the developed model was supplied with the predictors while the saved acquired patterns (support vectors) during training phase were implemented for predictions.

**Fig 6:**
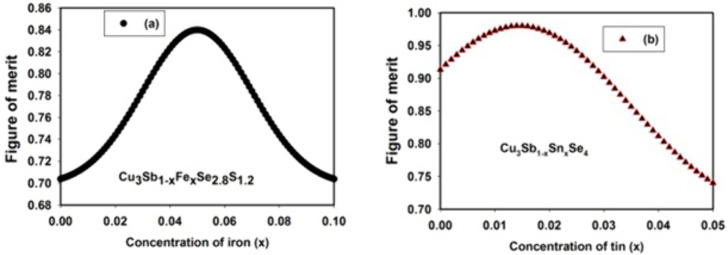
Effect of dopant substitution on thermoelectric figure of merit of (a) Cu_3_Sb_1-x_Fe_x_Se_2.8_S_1.2_ at a temperature of 600K and (b) Cu_3_Sb_1-x_Sn_x_Se_4_ at a temperature of 673 K.

For Cu_3_Sb_1-x_Fe_x_Se_2.8_S_1.2_ compound presented in **Fig 6a**, concentration of iron varied between 0 and 0.1 while the estimated thermoelectric figure of merits is shown as presented. Replacement of antimony (Sb) particles with iron (Fe) in the crystal structure of Cu_3_Sb_1-x_Fe_x_Se_2.8_S_1.2_ results into thermoelectric figure of merit enhancement up to the concentration of 0.05 after which further replacement lowers the thermoelectric figure of merit. Similarly, incorporation of tin (Sn) particles as substitutes to Sb in crystallographic structure of Cu_3_Sb_1-x_Sn_x_Se_4_ compound is presented in **Fig 6b**. Replacing Sb particles with Sn initially elevates thermoelectric figure of merit while further substitution continues to lower the values of thermoelectric figure of merit of the samples.

## 5. Conclusion

This work models the thermoelectric figure of merit of Cu_3_SbSe_4_ system of copper-based chalcogenides compound using hybrid genetic algorithm incorporated support vector regression (GESVR) and random forest regression (RFR) with ensemble of decision trees operational principle. The descriptors to the models include the temperature, ionic radius of inclusion at Cu-site, inclusion concentration at Cu-site, ionic radius of first inclusion at Sb-site, inclusion concentration at Sb-site, ionic radius of second inclusion at Sb-site, inclusion concentration at Sb-site, ionic radius of dopant at Se-site and concentration of dopant at Se-site. GESVR model outperforms RFR model for thermoelectric figure of merit prediction with respective improvement of 83.43%,3.38% and 85.45% using MAE, CC and RMSE metrics for training compounds of Cu_3_SbSe_4_ system of materials. For testing samples of Cu_3_SbSe_4_ system of materials, corresponding percentage improvement of 188.04%, 30.18% and 42.36%, were respectively obtained. The developed GESVR model demonstrates lower MAE as compared to RFR based model for the entire data samples. The developed GESVR model further models the influence of Fe and Sn particles inclusion in Cu_3_Sb_1-x_Fe_x_Se_2.8_S_1.2_ and Cu_3_Sb_1-x_Sn_x_Se_4_ compounds, respectively. The fact that the developed models utilize descriptors that can be easily fetched without any laboratory experiment coupled with the accuracy associated with the models would ultimately strengthen exploration of Cu_3_SbSe_4_ system of copper-based chalcogenides compound for electricity generation from wasted heat energy and ultimately address the global energy crisis. The developed models are limited to the prediction of energy conversion efficiency of Cu_3_SbSe_4_-based compounds. Other computational intelligence algorithms could be explored in future work for prediction of energy conversion efficiency of Cu_3_SbSe_4_-based compounds.

## References

[1] Rahman AA, Bhattacharya A, Sarma A. Synthesis of Cu 3 SbS 4, Cu 3 SbSe 4 and CuSbTe 2 thin films via chalcogenation of sputtered Cu-Sb metal precursors. Thin Solid Films. 2022;754(July 2021):2–8.

[2] Yin Z, et al. Synergistically optimized electron and phonon transport of polycrystalline BiCuSeO via Pb and Yb co-doping. Appl Mater Interfaces. 2021;13. 10.1021/acsami.1c19266 34817977

[3] Han G, Lu Y, Jia H, Ding Z, Wu L, Shi Y, et al. Magnesium-based energy materials: Progress, challenges, and perspectives. J Magnes Alloy. 2023;11(11):3896–925. doi: 10.1016/j.jma.2023.08.009

[4] Zhu X, Zuo Z, Wang W, Liu R, Jia B. Design and single/multi-objective optimization of N-type Skutterudite/P-type half-Heusler-based thermoelectric modules based on an improved thermal resistance model. Renew Energy. 2025;250:123206. doi: 10.1016/j.renene.2025.123206

[5] Zhu X, Zuo Z, Wang W, Jia B, Liu R. Parameter interaction analysis and comprehensive performance optimization of a thermoelectric generator system integrating a wide temperature range of thermoelectric modules. Energy Convers Manag. 2025;342(July):120027.

[6] Li A, Fu C, Zhao X, Zhu T. High-Performance Mg 3 Sb 2- x Bi x thermoelectrics: progress and perspective. Research. 2020;2020. 10.34133/2020/1934848 PMC7877388 33623901

[7] Zhang Y, Tian Y, Zhang Z, Jia Y, Zhang B, Jiang M, et al. Magnetic properties and giant cryogenic magnetocaloric effect in B-site ordered antiferromagnetic Gd2MgTiO6 double perovskite oxide. Acta Materialia. 2022;226:117669. doi: 10.1016/j.actamat.2022.117669

[8] Kumar A, Dhama P, Banerji P. Enhanced thermoelectric properties in Bi and Te doped p -type Cu3SbSe4 compound. AIP Conf Proc. 2018;1942.

[9] Li Y, Qin X, Li D, Li X, Liu Y, Zhang J, et al. Transport properties and enhanced thermoelectric performance of aluminum doped Cu3SbSe4. RSC Adv. 2015;5(40):31399–403. doi: 10.1039/c5ra02030a

[10] Wang B, Wang Y, Zheng S, Liu S, Li J, Chang S, et al. Improvement of thermoelectric properties of Cu3SbSe4 hierarchical with in-situ second phase synthesized by microwave-assisted solvothermal method. J Alloys Compd. 2019;806:676–82. doi: 10.1016/j.jallcom.2019.07.292

[11] Feng B, Li G, Pan Z, Hu X, Liu P, He Z. Enhanced thermoelectric performance in BiCuSeO oxyselenides via Ba/ Te dual-site substitution and 3D modulation doping. J Solid State Chem. 2018;266(July):297–303.

[12] Ren G-K, Lan J, Butt S, Ventura KJ, Lin Y-H, Nan C-W. Enhanced thermoelectric properties in Pb-doped BiCuSeO oxyselenides prepared by ultrafast synthesis. RSC Adv. 2015;5(85):69878–85. doi: 10.1039/c5ra13191j

[13] Mukhtar M, Munisa L, Saleh R. Co-precipitation synthesis and characterization of nanocrystalline zinc oxide particles doped with Cu2 ions. Mater Sci Appl. 2012;03(08):543–51.

[14] Zhang Y, et al. Enhancing thermoelectric performance in P-type Mg 3 Sb 2 -based Zintls through optimization of band gap structure and nanostructuring. J Mater Sci Technol. 2024;170:25–32.

[15] Christopher B, et al. Correlation between structural and transport properties of electron beam irradiated PrMnO3 compounds. Solid State Commun. 2018;270(November 2017):30–7.

[16] Lan J, et al. Doping for higher thermoelectric properties in p-type BiCuSeO oxyselenide. Appl Phys Lett. 2013;123905.

[17] Eslamifar M, Eghbali M. Nonlinear responses and optical limitation of copper nanoparticles by Z-scan method. J Opt Photon Res. 2024;1(3):145–50. doi: 10.47852/bonviewjopr42021706

[18] Loretz RA. Modified chalcogenide glass equations for the activation energy of crystallization. J Opt Photonics Res. 2024;1(November 2023):16–22.

[19] Li JM, et al. Realized high power factor and thermoelectric performance in Cu 3 SbSe 4. Intermetallics. 2019;109(December 2018):68–73.

[20] Kim D, Kim I-H. Thermoelectric performance of non-stoichiometric permingeatite Cu3+mSbSe4. Materials (Basel). 2024;17(17):4345. doi: 10.3390/ma17174345 39274733 PMC11396531

[21] Liu C, et al. Charge transfer engineering to achieve extraordinary power generation in GeTe-based thermoelectric materials. Sci Adv. 2023:1–10. 10.1126/sciadv.adh0713 PMC10132743 37126545

[22] Bhardwaj R, Bhattacharya A, Tyagi K, Gahtori B. Tin doped Cu 3 SbSe 4: A stable thermoelectric analogue for the mid- temperature applications. Mater Res Bull. 2019;113(December 2018):38–44.

[23] Zhou T, Wang L, Zheng S, Hong M, Fang T, Bai P. Self-assembled 3D flower-like hierarchical Ti-doped Cu 3 SbSe 4 microspheres with ultralow thermal conductivity and high zT. Nano Energy. 2018;49(April):221–9.

[24] Chang C, Chen C, Chiu W, Chen Y. Enhanced thermoelectric properties of Cu3SbSe4 by germanium doping. Mater Lett. 2017;186(October 2016):227–30.

[25] Zhao L, et al. Enhancing thermoelectric and mechanical properties of p-type Cu3SbSe4 -based materials via embedding nanoscale Sb2Se3. Mater Chem Phys. 2022;292(August):126669.

[26] Xie D, Zhang B, Zhang A, Chen Y, Yan Y, Yang H, et al. High thermoelectric performance of Cu3SbSe4 nanocrystals with Cu2-xSe in situ inclusions synthesized by a microwave-assisted solvothermal method. Nanoscale. 2018;10(30):14546–53. doi: 10.1039/c8nr03550d 30024012

[27] Zhang D, Yang J, Bai H, Luo Y, Wang B, Hou S, et al. Significant average ZT enhancement in Cu3SbSe4-based thermoelectric material via softening p–d hybridization. J Mater Chem A. 2019;7(29):17648–54. doi: 10.1039/c9ta05115e

[28] Murillo-escobar J, Sepulveda-suescun JP, Correa MA, Orrego-metaute D. Urban climate forecasting concentrations of air pollutants using support vector regression improved with particle swarm optimization: case study in Aburrá Valley, Colombia. Urban Clim. 2019;29(August 2018):100473.

[29] Owolabi TO. Modeling magnetocaloric effect of doped EuTiO3 perovskite for cooling technology using swarm intelligent based support vector regression computational method. Mater Today Commun. 2023;36:106688. doi: 10.1016/j.mtcomm.2023.106688

[30] Owolabi TO, Oloore LE, Akande KO, Olatunji SO. Modeling of magnetic cooling power of manganite-based materials using computational intelligence approach. Neural Comput Appl. 2018;30(1):1.

[31] Zhao W, Tao T, Zio E. System reliability prediction by support vector regression with analytic selection and genetic algorithm parameters selection. Appl Soft Comput J. 2015;30:792–802. doi: 10.1016/j.asoc.2015.02.026

[32] Roy A, Chakraborty S. Support vector regression based metamodel by sequential adaptive sampling for reliability analysis of structures. Reliab Eng Syst Saf. 2020;200(February):106948.

[33] Osuna E, Freund R, Girosit F. Training support vector machines: an application to face detection. In: Proceedings of IEEE Computer Society Conference on Computer Vision and Pattern Recognition. 1997. pp. 130–6.

[34] Owolabi TO, Akande KO, Olatunji SO. Development and validation of surface energies estimator (SEE) using computational intelligence technique. Comput Mater Sci. 2015;101:143–51.

[35] Olubosede O, Amiruddin M, Rahman A, Alqahtani M, Souiyah A, Mouftahou TOO, et al. Tailoring the energy harvesting capacity of zinc selenide semiconductor nanomaterial through optical band gap. Crystals. 2022;12(36):1–13.

[36] Shi Q, Niu G, Lin Q, Xu T, Li F, Duan Y. Quantitative analysis of sedimentary rocks using laser-induced breakdown spectroscopy: comparison of support vector regression and partial least squares regression chemometric methods. J Anal At Spectrom. 2015;30(12):2384–93. doi: 10.1039/c5ja00255a

[37] Khosravi A, Nahavandi S, Creighton D, Atiya AF. Comprehensive review of neural network-based prediction intervals and new advances. IEEE Trans Neural Netw. 2011;22(9):1341–56. 21803683 10.1109/TNN.2011.2162110

[38] Mosavi BC, Sajedi Hosseini F, Abdolshahnejad M, Gharechaee H, Dineva AA. Susceptibility prediction of groundwater hardness using ensemble machine learning models. Water. 2020;12:1–17.

[39] Oloore L, Owolabi T, Fayose S, Adegoke M, Akande K, Olatunji S. Modeling of semiconductors refractive indices using hybrid chemometric model. Model Meas Control A. 2018;91(3):95–103. doi: 10.18280/mmc_a.910301

[40] Qin T, Zeng S, Guo J. Robust prognostics for state of health estimation of lithium-ion batteries based on an improved PSO–SVR model. Microelectron Reliab. 2015;55(9–10):1280–4. doi: 10.1016/j.microrel.2015.06.133

[41] Suleiman MA, Owolabi TO, Adeyemo HB, Olatunji SO. Modeling of autoignition temperature of organic energetic compounds using hybrid intelligent method. Process Saf Environ Prot. 2018;120:79–86.

[42] Ansari HR, Gholami A. An improved support vector regression model for estimation of saturation pressure of crude oils. Fluid Phase Equilibria. 2015;402:124–32. doi: 10.1016/j.fluid.2015.05.037

[43] Schmitt LM. Theory of genetic algorithms. Theor Comput Sci. 2001;259(1–2):1–61.

[44] Owolabi TO, Amiruddin M, Rahman A. Prediction of band gap energy of doped graphitic carbon nitride using genetic algorithm-based support vector regression and extreme learning machine. 2021:1–17.

[45] Kumar A, Bhowmik D, Pal J, Sen P. Towards the realization of regular clocking-based QCA circuits using genetic algorithm ✩. Comput Electr Eng. 2022;97(November 2021):107640.

[46] He Y, Wu C, Fan Y. Exploring the drivers of local government budget coordination: a random forest regression analysis. Int Rev Econ Financ. 2024;93(PA):1104–13.

[47] Zhang T, Liang L, Wang K, Tang H, Yang X, Duan Y, et al. A novel approach for the quantitative analysis of multiple elements in steel based on laser-induced breakdown spectroscopy (LIBS) and random forest regression (RFR). J Anal At Spectrom. 2014;29(12):2323–9. doi: 10.1039/c4ja00217b

[48] Ahmad MW, Reynolds J, Rezgui Y. Predictive modelling for solar thermal energy systems: A comparison of support vector regression, random forest, extra trees and regression trees. J Clean Prod. 2018;203:810–21.

[49] Zhao L, Yang J, Zou Y, Hu J, Liu G, Shao H, et al. Tuning Ag content to achieve high thermoelectric properties of Bi-doped p-type Cu3SbSe4-based materials. J Alloys Compd. 2021;872:159659. doi: 10.1016/j.jallcom.2021.159659

[50] Wang B, et al. Synergistic modulation of power factor and thermal conductivity in Cu 3 SbSe 4 towards high thermoelectric performance. Nano Energy. 2020;71(February):104658.

[51] Wang B, Zheng S, Chen Y, Wang Q, Li Z, Wu Y, et al. Realizing ultralow thermal conductivity in Cu3SbSe4 via all-scale phonon scattering by co-constructing multiscale heterostructure and IIIB element doping. Mater Today Energy. 2021;19:100620. doi: 10.1016/j.mtener.2020.100620

[52] Zhao L, et al. Enhanced figure of merit for famatinite Cu 3 SbSe 4 via band structure tuning and hierarchical architecture. J Mater. 2023;9.

[53] Zhang D, Yang J, Jiang Q, Fu L, Xiao Y, Luo Y, et al. Improvement of thermoelectric properties of Cu 3 SbSe 4 compound by In doping. Mater Des. 2016;98:150–4. doi: 10.1016/j.matdes.2016.03.001

[54] Wei S, et al. Enhancing the thermoelectric and mechanical properties of Cu 3 SbSe 4 -based materials by defect engineering and covalent bonds reinforcement. J Alloys Compd. 2024;997(March).

[55] Wei S, et al. Enhancing the effective mass and covalent bond strength of Cu 3 SbSe 4 -based thermoelectric materials by Mn/ Sn co-doping. Mater Today Phys. 2023;38(September).

[56] Pal A, et al. Enhancement of low-temperature thermoelectric performance via Pb doping in Cu 3 SbSe 4. J Phys Chem Solids. 2023;175(December 2022):111197.

[57] Wei S, et al. Enhanced thermoelectric properties of Cu 3 SbSe 4 -based materials by synergistic modulation of carrier concentration and phonon scattering. J Mater. 2024;10.

[58] Kumar A, Dhama P, Banerji P. Effect of Cu deficiency on the transport behavior and thermoelectric properties in Cu3SbSe4. AIP Conf Proc. 2017;1832:1–4.

[59] You A, Be M, In I. Enhanced electrical transport and thermoelectric properties in Ni doped Cu3SbSe4. AIP Conf Proc. 2020;050030:8–12.

[60] Li D, Li R, Qin X-Y, Song C-J, Xin H-X, Wang L, et al. Co-precipitation synthesis of nanostructured Cu3SbSe4 and its Sn-doped sample with high thermoelectric performance. Dalton Trans. 2014;43(4):1888–96. doi: 10.1039/c3dt52447g 24264386

[61] Li D, Li R, Qin X-Y, Zhang J, Song C-J, Wang L, et al. Co-precipitation synthesis of Sn and/or S doped nanostructured Cu3Sb1−xSnxSe4−ySy with a high thermoelectric performance. CrystEngComm. 2013;15(36):7166. doi: 10.1039/c3ce40956b

[62] Chen K, Di Paola C, Du B, Zhang R, Laricchia S, Bonini N, et al. Enhanced thermoelectric performance of Sn-doped Cu3SbS4. J Mater Chem C. 2018;6(31):8546–52. doi: 10.1039/c8tc02481b

